# Ex vivo machine perfusion: current applications and future directions in liver transplantation

**DOI:** 10.1007/s00423-020-02014-7

**Published:** 2020-11-20

**Authors:** Julian Michelotto, Joseph M. G. V. Gassner, Simon Moosburner, Vanessa Muth, Madhukar S. Patel, Markus Selzner, Johann Pratschke, Igor M. Sauer, Nathanael Raschzok

**Affiliations:** 1 Charité – Universitätsmedizin Berlin, Department of Surgery, Experimental Surgery, corporate member of Freie Universität Berlin, Humboldt- Universität zu Berlin and Berlin Institute of Health, Campus Charité Mitte | Campus Virchow-Klinikum, Berlin, Germany; 2 Department of Surgery, Abdominal Transplant and HPB Surgery, Ajmera Family Transplant Centre, Toronto General Hospital, Toronto, ON Canada

**Keywords:** Machine perfusion, Liver transplantation, Clinical application, Systematic literature review

## Abstract

**Background:**

Liver transplantation is the only curative treatment option for end-stage liver disease; however, its use remains limited due to a shortage of suitable organs. In recent years, ex vivo liver machine perfusion has been introduced to liver transplantation, as a means to expand the donor organ pool.

**Purpose:**

To present a systematic review of prospective clinical studies on ex vivo liver machine perfusion, in order to assess current applications and highlight future directions.

**Methods:**

A systematic literature search of both PubMed and ISI web of science databases as well as the ClinicalTrials.gov registry was performed.

**Results:**

Twenty-one articles on prospective clinical trials on ex vivo liver machine perfusion were identified. Out of these, eight reported on hypothermic, eleven on normothermic, and two on sequential perfusion. These trials have demonstrated the safety and feasibility of ex vivo liver machine perfusion in both standard and expanded criteria donors. Currently, there are twelve studies enrolled in the clinicaltrials.gov registry, and these focus on use of ex vivo perfusion in extended criteria donors and declined organs.

**Conclusion:**

Ex vivo liver machine perfusion seems to be a suitable strategy to expand the donor pool for liver transplantation and holds promise as a platform for reconditioning diseased organs.

## Introduction

Liver transplantation is the only curative treatment option for acute liver failure and advanced hepatobiliary malignancies. Unfortunately, this therapy is limited by the supply of suitable donor livers for transplantation [1], as reflected in high waitlist mortality [2]. Despite much progress in transplantation, static cold storage (SCS) remains the standard technique for graft preservation after procurement, as it reduces the sequelae of ischemic injury [3, 4].

To ameliorate the gap between supply and demand for liver transplantation, the use of marginal livers has increased [5–10]. Marginal liver grafts may be obtained from elderly donors, those with clinical factors predisposing them to hepatic steatosis, or from donation after circulatory death (DCD), compared with traditional donation after brain death (DBD) [11–15]. Elderly donors, most often defined as originating from donors above the age of 60 [16, 17], run a risk of impaired metabolic function and cellular regeneration due to a life-long exposure to hepatotoxic agents and fibrotic remodeling [18]. With regard to hepatic steatosis, it is defined by the presence of triglyceride droplets in more than 5% of hepatocytes of either small (microvesicular) or large (macrovesicular) composition [19]. While grafts affected by mild macrovesicular steatosis (< 30%) are considered suitable for transplantation [20], moderate to severe steatosis was revealed as independent prognostic factor for poor postoperative outcomes [21, 22]. Although the pathophysiologic mechanisms are not completely understood, these fat deposits are often a result of genetic predisposition combined with high calorie intake, alcohol abuse, or old age [23, 24]. Hepatic steatosis and fibrotic remodeling of the liver are hypothesized to lead to decreased metabolic function, obstructed microvascular perfusion, and a greater susceptibility of ischemia-reperfusion injury (IRI) after transplantation [25, 26]. Lastly, DCD organs are retrieved after a period of reduced and ultimately stopped circulation, inherently resulting in increased warm ischemia until flushing of the organ with cold perfusion solution. Depending on the preexisting condition of the organ, this may lead to distinct cellular injury and ischemia-related damage of non-parenchymal cells such as Kupffer cells and cholangiocytes. Taken together, the use of marginal donor grafts is associated with an increased risk of clinically significant postoperative complications such as early allograft dysfunction (EAD), primary nonfunction (PNF), or ischemic cholangiopathy [11, 27–34].

**Fig. 1:**
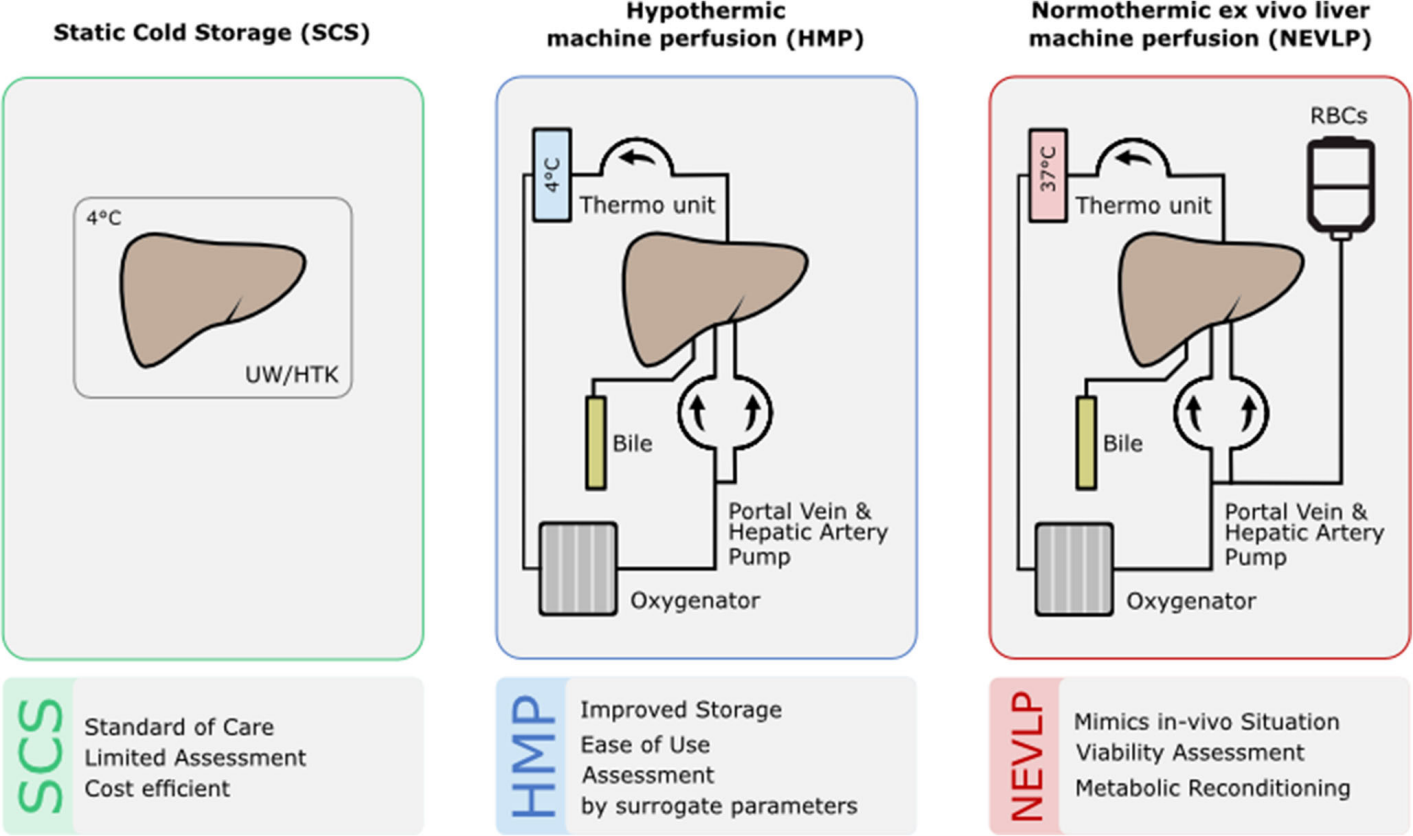
Preservation methods for liver transplantation with static cold storage (SCS), hypothermic machine perfusion (HMP), and normothermic ex vivo liver machine perfusion (NEVLP) and their respective advantages

Historically, liver machine perfusion had been considered for organ preservation, but early approaches were not widely adopted. Recently, with much technological progress being made, there is a renewed interest to implement this technique to not only assess, but also to improve the quality of liver grafts (Fig.1) [35–37].

As such, one of the primary goals of ex vivo liver machine perfusion is to mitigate the effects of graft IRI [38, 39]. Through perfusion at different temperatures, ranging from hypothermic (+ 4 to + 10 °C) to subnormothermic (+ 15 °C to 30 °C) and to normothermic (37 °C) [40], graft metabolites can be flushed, nutrient supply optimized, and microvascular circulation maintained. [3, 4, 41, 42]. In the last decade, several studies ranging from proof-of-concept animal experiments to randomized trials have been published on the different modes of ex vivo liver machine perfusion. The aim of this systematic review is to provide a summary of the literature on ex vivo liver machine perfusion, with a focus on current clinical application and an outlook on future directions in transplantation. Mechanistic aspects of liver machine perfusion and experimental studies in animal models were thus excluded as they were outside the scope of this review.

## Methods

### Search strategy

**Fig. 2:**
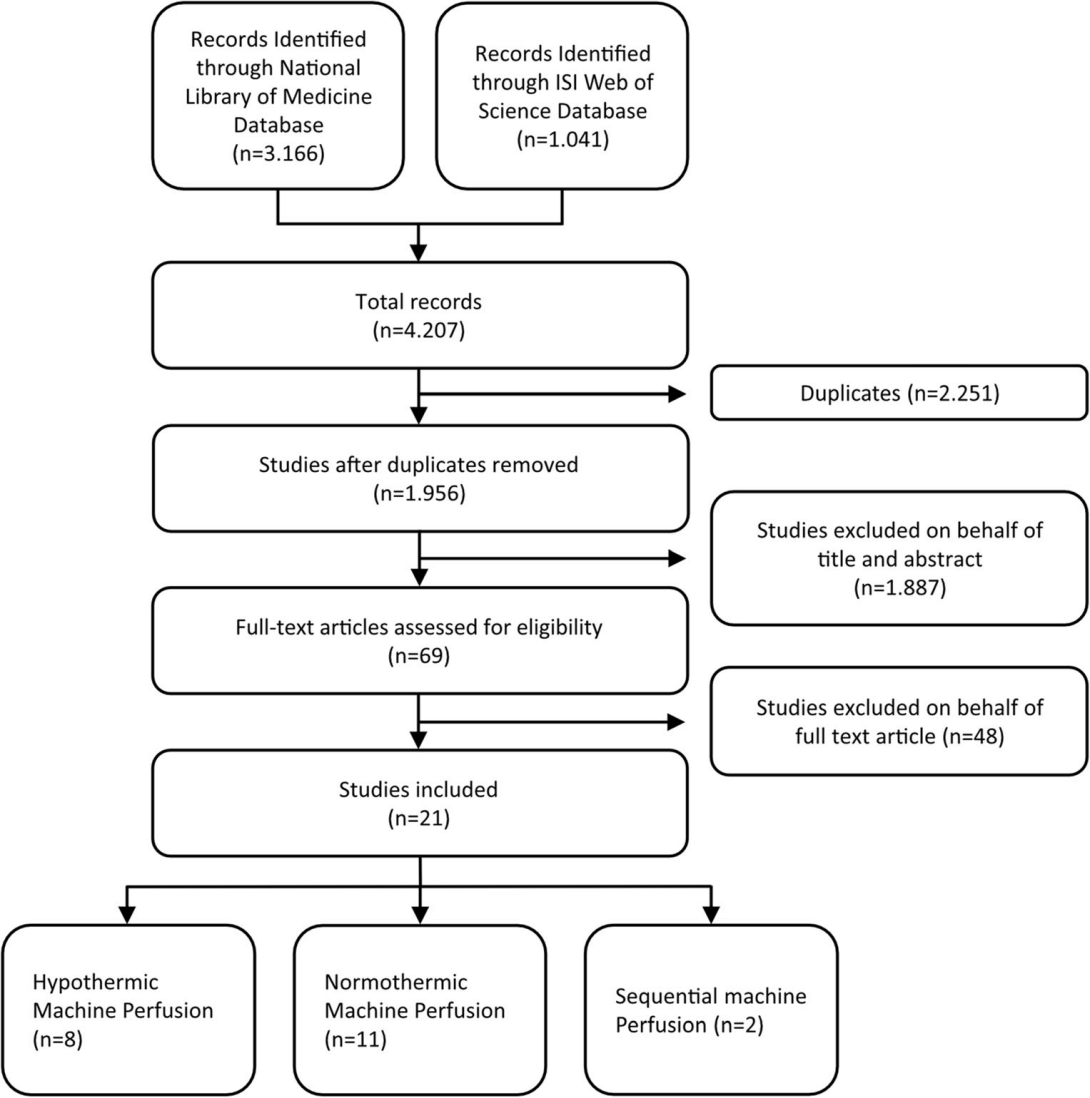
Flowchart of the performed systematic literature research

A systematic literature review was performed following the Preferred Reporting Items for Systematic reviews and Meta-Analyses for Protocols 2015 (PRISMA-P) (Fig. 2). A wide-ranging screening of the National Library of Medicine Database and the ISI Web of Science Database was performed on April 7, 2020, and last updated on June 18, 2020, in order to identify literature on liver preservation using machine perfusion (MP) as an alternative to SCS in human orthotopic liver transplantation.

The following search queries were performed:

1. “liver AND machine AND perfusion AND human”
2. “liver transplantation AND perfusion AND human”
3. “liver transplantation AND perfusion AND CLINICAL TRIAL”
4. “liver transplantation AND normotherm* AND human”
5. “liver transplantation AND hypotherm* AND human”

Additionally, the ClinicalTrials.gov registry of the US National Library of Medicine was searched on April 9, 2020, for the following MeSH terms:

“Machine Perfusion AND Liver Transplantation”

### Inclusion criteria

Articles meeting inclusion criteria for this review were published prospective studies using any type of ex vivo machine perfusion device for organ preservation in liver transplantation. Editorials, letters, reviews, case reports, conference abstracts, and video articles were excluded. As were follow-up studies on already published grafts, perfusate samples, or liver biopsies which reported no further information on peri- or postoperative outcomes of the respective recipients. With regard to clinical trials, inclusion criteria involved any kind of trials on transplantation of liver grafts after ex vivo machine perfusion with the following recruitment statuses: “recruiting,” “active, not recruiting,” “not yet recruiting,” or “enrolling by invitation.”

### Data extraction

A two-stage independent screening method was applied by two of the authors (JM and JG). In case of discordance, the corresponding author NR was consulted, and consensus was made via discussion. During stage one of data extraction, the titles and abstracts of all retrieved records were reviewed and unsuitable studies were excluded. During stage two, full text articles of remaining studies were read carefully and assessed for inclusion criteria. For the identification of clinical trials, the study description was reviewed by NR and MP and results discussed with the remaining authors. Extracted data were reviewed and analyzed by all authors.

## Results

### General information

**Fig. 3:**
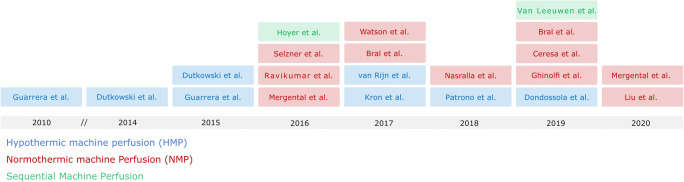
Time line of published clinical trials involving liver machine perfusion, color-coded according to applied mode of perfusion with hypothermic machine perfusion (HMP, blue), normothermic ex vivo liver machine perfusion (NEVLP, red) and sequential machine perfusion (green)

Systematic literature search of the National Library of Medicine database and the ISI Web of Science database identified 1.956 unique records. Based on to title and abstract, 1.887 papers could be removed, leaving 69 articles for full text analysis. Of these, 48 publications did not meet inclusion criteria. Two of the identified publications were video-articles and therefore excluded. One publication using hypothermic oxygenated perfusion (HOPE) was removed given that its primary focus was on the evaluation of the safety and feasibility of normothermic regional perfusion prior to HOPE in DCD donors with extended warm ischemic times rather than the assessment of this technique for the purpose of preservation. Further five studies were identified as follow-up studies on perfusate, liver or bile duct samples of pre-published clinical trials and consequently excluded. Taken together, 21 publications on prospective clinical trials using ex vivo liver machine perfusion for graft preservation or graft assessment in liver transplantation were identified (Fig. 3). Eight reported on hypothermic (Table 1), eleven on normothermic (Table 2), and two on sequential perfusion (Table 3). Of note, no clinical trial was identified evaluating the use of sub-normothermic machine perfusion.

**Table 1:** Prospective clinical trials on hypothermic ex vivo liver machine perfusion

Publication	Perfusion device	Donors	RCT	*N*	Group	Donor age	Recipient age	Recipient MELD	Perfusion time (min)	Peak AST (POD1-7) (U/l)	EAD (%)	ICU (d)	HLS (d)	1-year patient survival (%)	1-year graft survival (%)	Biliary complication
Guarrera et al. (2010)	Medtronic PBS	DBD	No	20	pSCS HMP	39.4 (± 2.5)	55.4 (± 6.2)	17.2 (± 7.4)	180–420	1154 (± 79.5)	5	n.a.	10.9 (± 4.7)	90	90	2
				20	SCS	45.6 (± 2.1)	52.7 (± 8.9)	16.8 (± 6.8)	-	3339 (± 755.1)	25	n.a.	15.3 (± 4.9)	90	90	4
Guarrera et al. (2015)	Medtronic PBS	n.a.	No	31	pSCS HMP	57.5 (± 17.8)	57.5 (± 8.0)	19.5 (± 5.9)	228 (± 54)	n.a.	19	n.a.	13.64 (± 10.9)	83,8	81	4
				30	SCS	57.9 (± 16.9)	58.4 (± 9.6)	21.4 (± 6.3)	-	n.a.	30	n.a.	20.14 (± 11.12)	80	80	13
Dutkowski et al. (2014)	Liver Assist	DCD	No	8	HOPE (DCD)	54 (37–64)	60 (50–67)	12 (6–23)	118 (104–160)	n.a.	n.a.	2	16,5	n.a.	n.a.	2
				8	SCS (DBD)	52 (26–66)	60 (52–70)	16 (7–27)	-	n.a.	n.a.	4,5	18	n.a.	n.a.	2
Dutkowski et al. (2015)	Liver Assist	DCD	No	25	HOPE (DCD)	54 (36–63)	60 (57–64)	13 (9–15)	118 (101–149)	1808 (1133–3547)	20	3 (1.3–5.7)	20 (14–23)	n.a.	90	5
				50	SCS (DCD)	48 (33–51)	56 (49–59)	16 (10–21)	-	2848 (1485–6724)	44	3 (2–6)	18 (15–29)	n.a.	69	23
				50	SCS (DBD)	59 (46–70)	54 (50–62)	11 (9–17)	-	1473 (762–3764)	22	3 (2–6)	17.5 (13–26)	n.a.	96	12
Kron et al. (2017)	Liver Assist	DBD/DCD	No	6	HOPE	65 (55–86)	55 (50–60)	11 (6–16)	138 (108–288)	n.a.	n.a.	3 (2–6)	n.a.	100	100	n.a.
				12	SCS	59 (54–70)	54 (42–73)	19 (6–34)	–	n.a.	n.a.	15 (1–84)	n.a.	42	n.a.	n.a.
van Rijn et al. (2017)	Liver Assist	DCD	No	10	pSCS D-HOPE	53 (47–57)	57 (54–62)	16 (15–22)	126 (123–135)	n.a.	n.a.	2 (2–6)	22 (16–33)	100	100	4
				20	SCS	53 (47–58)	52 (42–60)	22 (17–27)	-	n.a.	n.a.	2 (1–5)	22 (15–32)	85	67	11
Dondossola et al. (2019)	Liver Assist	DBD/DCD	No	6	pSCS D-HOPE	51 (36–72)	59 (47–66)	17 (12–35)	240 (180–320)	1278 (577–8304)	33	2 (1–9)	16 (11–23)	n.a.	n.a.	0
Patrono et al. (2018)	Liver Assist	DBD	No	4	pSCS D-HOPE	70 (54–82)	52 (25–61)	14.5 (7–35)	176 (150–200)	685 (72–2473)	50	4 (2–22)	12 (8–46)	n.a.	n.a.	0

**Table 2:** Prospective clinical trials on normothermic ex vivo liver perfusion

Publication	Perfusion device	Donors	RCT	*N*	Group	Donor age	Recipient age	Recipient MELD	Perfusion time (min)	Peak AST (POD 1–7) (U/l)	EAD (%)	ICU (d)	HLS (d)	1-year patient survival (%)	1-year graft survival (%)	Biliary complication
Ravikumar et al. (2016)	OrganOX	DBD/DCD	No	20	NMP	58 (21–85)	54.4 (33–66)	12 (7–27)	558 (210–1110)	417 (84–4681)	15	3 (1–8)	12 (6–34)	95	n.a.	3
				40	Control (SCS)	58.5 (21–82)	55.0 (27–65)	14 (6–25)	-	902 (218–8786)	22.5	3 (1–41)	14 (8–88)	n.a.	n.a.	n.a.
Selzner et al. (2016)	OrganOX	DBD/DCD	No	10	NMP	48 (17–75)	56 (45–71)	21 (8–40)	480 (340–580)	1182 (167–6700)	n.a.	1 (0–8)	11 (8–17)	n.a.	n.a.	0
				30	Control (SCS)	46 (22–68)	54 (42–63)	23 (7–37)	-	1474 (521–5156)	n.a.	2 (0–23)	13 (7–89)	n.a.	n.a.	n.a.
Bral et al. (2017)	OrganOX	DBD/DCD	No	10	NMP	56 (14–71)	53 (28–67)	13 (9–32)	690 (198–1350)	1252 (383–2600)	55.5	16 (2–65)	45 (13–114)	n.a.	n.a.	0
				30	Control (SCS)	52 (20–77)	59 (43–69)	19 (7–34)	-	839 (153–2600)	29.6	4 (1–29)	25 (9–89)	n.a.	n.a.	4
Nasralla et al. (2018)	OrganOX	DBD/DCD	Yes	121	NMP	56 (16–84)	55 (20–72)	13 (6–35)	547.5 (85–1388)	488.1 (409–583)	10,1	4 (2–7)	15 (10–24)	95.8 (90–98)	95.0 (89–98)	13
				101	Control (SCS)	56 (20–86)	55 (22–70)	14 (6–29)	-	964.9 (795–1172)	29,9	4 (3–7)	15 (11–24)	97 (91–99)	96 (90–99)	15
Liu et al. (2020)	Self-made	DBD/DCD	No	21	NMP/pSCS NMP	35.0 (± 12.7)	57.0 (± 7.1)	19.1 (± 7.7)	296 (201–472)	1357 (± 1492)	19	2.5 (± 2.8)	13.4 (± 9.1)	95.24	100	n.a.
				84	control (SCS)	34.8 (± 15.0)	57.4 (± 8.4)	19.4 (± 8.7)	-	2615 (± 2541)	46	2.7 (± 3.7)	15.7 (± 14.2)	n.a.	n.a.	n.a.
Bral et al. (2019)	OrganOX	DBD/DCD	No	17	NMP	40 (14–71)	59 (50–63)	25 (21–32)	618 (198–1344)	709 (283–1921)	35	6 (3–48)	43 (22–61)	n.a.	n.a.	4
				26	pSCS NMP	37 (15–67)	57 (40–63)	22 (17–24)	468 (249–1008)	863 (460–1640)	19	2 (2–4)	16 (12–20)	n.a.	n.a.	4
Ceresa et al. (2019)	OrganOx	DBD/DCD	No	31	pSCS NMP	58 (17–78)	58 (25–73)	14 (7–24)	504 (± 244)	457 (92–8669)	13	3 (1–20)	3 (7–31)	90	84	2
				104	NMP	55 (17–83)	56 (20–73)	13 (6–33)	n.a.	465 (68–8822)	11	4 (1–23)	14 (10–24)	n.a.	94	n.a.
Ghinolfi et al. (2019)	Liver Assist	DBD	Yes	10	pSCS NMP	81 (77–87)	57 (46–61)	12.5 (9–16)	250 (195–282)	709 (371–1575)	20	n.a.	17 (14–22)	n.a.	n.a.	1
				10	SCS	80 (72–87)	55 (43–61)	9.5 (8–15)	-	574 (377–1162)	10	n.a.	12 (11–15)	n.a.	n.a.	0
Mergental et al. (2016)	Liver Assist/OrganOx	DBD/DCD	No	5	pSCS NMP	49 (29–54)	56 (47–66)	8 (7–17)	332 (318–564)	n.a.	0	3 (2–6)	10 (6–15)	n.a.	n.a.	-
Watson et al. (2017)	Liver Assist	DBD/DCD	No	12	pSCS NMP	56 (24–67)	57 (46–65)	17 (10–26)	284 (122–530)	n.a.	n.a.	n.a.	n.a.	92	83	3
				24	SCS	54 (22–72)	n.a.	n.a.	–	n.a.	n.a.	n.a.	n.a.	96	88	7
Mergental et al. (2020)	OrganOx	DBD/DCD	No	22	pSCS NMP	56 (45–65)	56 (46–65)	12 (9–16)	587 (450–705)	n.a.	31.8	3.5 (3–4)	10 (8–17)	100	86.4	6
				44	SCS	n.a.	n.a.	n.a.	-	n.a.	9.1	2.0 (1–5)	9 (8–11)	95.5	86,4	4

**Table 3:** Prospective clinical trials on sequential ex vivo liver perfusion

Publication	Perfusion device	Donors	RCT	*N*	Group	Donor age	Recipient age	Recipient MELD	Perfusion time (min)	Peak AST (POD 1-7) (U/l)	EAD (%)	ICU (d)	HLS (d)	1-year patient survival (%)	1-year graft survival (%)	Biliary complication
Hoyer et al. (2016)	Liver Assist	DBD	No	6	COR	58.5 (51–71)	52.5 (43–65)	18 (11–23)	90	n.a.	0	3 (2–26)	21.5 (14–103)	n.a.	n.a.	n.a.
				106	SCS	66.5 (42–88)	55 (21–70)	13 (6–28)	-	n.a.	35.9	21.5 (0–161)	19 (0–168)	n.a.	n.a.	n.a.
van Leeuwen et al. (2019)	Liver Assist	DCD	No	11	Perfusion (DCD)	63 (52–72)	61 (55–66)	14 (13–15)	n.a.	751 (483–1757)	n.a.	n.a.	n.a.	100	100	1
				24	SCS (DCD)	52 (48–56)	56 (48–61)	18 (11–24)	-	2406 (1102-4573)	n.a.	n.a.	n.a.	88	80	4
				36	SCS (DBD)	61 (47–66)	52 (40–60)	15 (10–22)	-	1646 (627–2494)	n.a.	n.a.	n.a.	100	100	3

With regard to currently registered studies, query of ClinicalTrials.gov identified a total number of 22 trials, out of which 12 met the inclusion criteria (Table 4). One clinical trial was excluded as it was primarily focused on developing a viability index for liver machine perfusion. The other nine trials were either completed or had unknown status. Out of the active studies, four were focused on hypothermic liver machine perfusion, while seven addressed normothermic machine perfusion. Sequential hypo- and normothermic perfusion is being evaluated in one ongoing trial.

**Table 4:** Current clinical trials on ex vivo liver machine perfusion

Title	Status	Features	Identifier
Hypothermic machine perfusion			
Study to evaluate performance of LifePort® Liver Transporter System, a machine perfusion system, for liver transplant	Recruiting	Regular organs, randomized, multicenter	NCT 03484455
Hypothermic oxygenated perfusion for extended criteria donors in liver transplantation (HOPExt)	Recruiting	ECD organs, DBD, randomized, multicenter	NCT 03929523
Post-static cold storage hypothermic oxygenated perfusion in Bergamo Liver Transplant Program	Recruiting	ECD organs, DBD and DCD, observational, single-center	NCT 03098043
Clinical trial of new hypothermic oxygenated perfusion system versus static cold storage	Recruiting	ECD organs, DBD, new HOPE device vs. static cold storage, randomized, single-center	NCT 03837197
Normothermic machine perfusion			
Normothermic liver preservation	Active, not yet recruiting	Regular organs, DBD and DCD, randomized, multicenter	NCT 02775162
Safety and feasibility of normothermic machine perfusion to preserve and evaluate orphan livers	Not yet recruiting	Declined organs, DBD and DCD. single group, single-center	NCT 03456284
Pilot study to assess safety and feasibility of normothermic machine preservation in human liver transplantation	Active, not recruiting	ECD (> 70 years), DBD and DCD, non-randomized	NCT 02515708
Normothermic liver preservation trial	Recruiting	ECD, DBD and DCD, single-center, non-randomized	NCT 03089840
Using ex vivo normothermic machine perfusion with the OrganOx Metra™ device to store human livers for transplantation	Enrolling by invitation	ECD, DBD and DCD mix, non-randomized	NCT 02478151
Viability testing and transplantation of marginal livers	Active, not recruiting	Declined organs, DBD and DCD, single-center, non-randomized	NCT 02740608
Efficacy of ex situ normothermic perfusion versus Cold storage in the transplant with steatotic liver graft	Recruiting	DBD and living donors, 30% - 60% of macrovesicular steatosis, single-center, randomized	NCT 03930459
Sequential perfusion			
Sequential hypo- and normothermic perfusion to preserve extended criteria donor livers for transplantation	Not yet recruiting	ECD, DBD and DCD, single group, single-center	NCT 04023773

### Hypothermic machine preservation

Based on perfusion technique, such as single- versus double-vessel perfusion and whether active oxygenation of the perfusate is performed, hypothermic machine preservation can be categorized as follows:

- HMP: hypothermic machine perfusion
- HOPE: hypothermic oxygenated machine perfusion
- D-HOPE: dual hypothermic oxygenated machine perfusion

In clinically used HMP and D-HOPE, liver grafts are perfused throughout the portal vein and the hepatic artery simultaneously, classifying them as double-vessel systems [43–47]. A single vessel approach is followed in HOPE, with organ perfusion exclusively via the portal vein [48–50]. While in HOPE and D-HOPE active oxygenation of the perfusate is performed [43–45, 48–50], it is omitted in HMP [46, 47].

The first prospective clinical trial investigating the role of HMP in human liver transplantation was reported by Guarrera et al. In 2010 [46], a total of 20 patients receiving standard liver grafts after HMP were compared with a matched cohort of patients undergoing liver transplantation after SCS. Following the nomenclature proposal for ex vivo liver machine perfusion published by Karangwa et al., the approach used can be classified as post-static cold storage HMP (pSCS-HMP), as HMP was initiated after graft arrival in SCS at the study center, with initial SCS times exceeding 3 hours [40]. For hypothermic dual vessel perfusion, a modified Medtronic Portable Bypass System was used, creating continuous flow through the hepatic artery and the portal vein. Although no active oxygenation of the perfusate (Vasosol®) was performed, the authors reported stable oxygen pressures throughout the perfusion (mean 137.2 ± 4.8 mmHg). Within this study, no case of PNF occurred; EAD was observed in 1 patient in the HMP group and in 5 patients in control group (*p* = 0.08). One-year patient and graft survival were 90% in both groups, with no death related to graft function. However, the HMP group had significantly shorter hospital length of stay (HLS) (*p* = 0.006), and significantly lower levels of post-operative peak aspartate aminotransferase (AST) (*p* = 0.011), peak alanine aminotransferase (ALT) (*p* = 0.044), total bilirubin (*p* = 0.042), and serum creatinine (*p* = 0.013) [46]. After safety and feasibility was shown, the group expanded their inclusion criteria on investigating the role of pSCS-HMP in the transplantation of ECD grafts: in 2015, Guarrera et al. published the outcomes of 31 “orphan” ECD grafts transplanted after pSCS-HMP compared with a cohort of matched SCS liver grafts [47]. Included organs were defined as ECD by either donor age above 65 years, hepatitis C virus positivity with 15% macrosteatosis, greater than 25% macrovesicular steatosis by biopsy, or evidence of significant donor ischemic injury (donor serum AST or ALT > 1000 IU/L) at the time of organ offer. Additionally, the term “orphan” was coined, to indicate that included grafts were either declined by all centers of their originating UNOS Region, or by all centers of the UNOS Region 9, except the study center. The study reported the occurrence of PNF in one patient of the HMP group and in two patients of the control group (*p* = 0.612), with 6 cases of EAD observed in the HMP group and 9 cases in the control subjects (*p* = 0.384). Three cases of vascular complications occurred in the HMP group and two cases in the SCS group, with a 1-year survival of 83.8% and 80.0%, respectively. Concerning biliary complications, significantly less cases were observed in the HMP-group (*p* = 0.001). Subgroup analyses showed that especially the occurrence of biliary strictures, as a cause for a biliary complication, differed between groups (*p* = 0.031). Patients in the HMP group had a significantly shorter HLS (*p* = 0.001), with lower peak serum ALT on post-operative day (POD) 1 (*p* = 0.049) and lower serum creatinine on POD 5 (*p* = 0.02) [47]. No adverse events due to perfusion device malfunction occurred in the reported studies [46, 47].

HOPE was first introduced into clinical practice in 2014, as a series of 8 DCD liver transplantations published by Dutkowski et al. [48]. The initial trial was performed as an initiative to reintroduce DCD at the University Hospital of Zürich (Switzerland), after an almost decade long hiatus of law changes. Starting with the first available DCD liver graft, a preservation protocol including HOPE was applied, with the intention to prevent adverse outcomes. Using an Organ Assist® ECOPS device, grafts were perfused solely via the portal vein with oxygenated UW gluconate solution at pressures below 3 mmHg. Six-month graft survival was 100%, with no case of PNF, delayed graft function, intrahepatic biliary cholangiopathy, or hepatic artery thrombosis, although two cases of extrahepatic biliary complications were observed [48]. For better evaluation of the impact of HOPE in DCD liver grafts, an international-matched case analysis was published by Dutkowski et al. the following year, comparing the outcomes of *n* = 25 HOPE-treated DCD liver grafts with a matched cohort of *n* = 50 SCS DCD and *n* = 50 SCS DBD liver transplants [49]. Transplantation of HOPE-treated grafts was once again performed at the University Hospital of Zürich, while data of SCS DCD transplantations was obtained from the transplantation programs of Rotterdam (NL) and Birmingham (UK). Primary endpoints of the study included the incidence and severity of biliary complications within the first year after transplantation. Secondary endpoints were related to liver IRI and function and graft survival. Total cold preservation time reported was significantly shorter in the HOPE group (*p* = 0.002). Comparing post-operative outcomes, HOPE-treated DCD grafts developed less cases of EAD (*p* = 0.046) and showed decreased levels of peak AST (*p* = 0.04), peak ALT (*p* = 0.02), and peak Bilirubin (*p* = 0.016) compared with SCS DCD controls. In the HOPE group, no case of PNF occurred. Regarding extrahepatic biliary complications, no differences were observed between groups, although significantly less cases of intrahepatic cholangiopathy were noted in HOPE-treated DCD grafts (*p* = 0.013). Overall, 1-year graft survival was 90% in HOPE-treated, compared with 69% in SCS DCD livers (*p* = 0.035) [49]. Additionally, outcomes were compared with a matched cohort of 50 DBD SCS liver transplantations, which showed no significant differences across the analyzed endpoints [49].

A third study applying HOPE, also performed by the Zürich group, was published in 2017: Kron et al. reported a series of 6 liver transplantations using HOPE-treated, steatotic liver grafts [50]. This pilot trial was initiated to evaluate promising observations made in rodent experiments published within the same article. Grafts had a median macrovesicular steatosis of 30% (20–30) with 5 livers being retrieved from DCD donors. Recipient Lab-MELD score ranged from 6 to 16. [50]. There were no cases of PNF and all patients were alive at 1-year follow-up. Compared with a cohort of DBD SCS steatotic grafts, matched for donor and recipient age as well as total preservation time, HOPE treatment showed lower ALT post reperfusion (*p* = 0.04) with higher rates of 1-year patient survival (*p* = 0.04) [50].

The first study evaluating end-ischemic D-HOPE in human DCD liver transplantation was published 2017 by Van Rijn et al. from the Netherlands [45]. The authors reported a series of 10 patients undergoing liver transplantation of DCD grafts treated with end-ischemic D-HOPE. In the trial, a Liver Assist (Organ Assist, Groningen, the Netherlands) device was used for pulsatile perfusion of the hepatic artery and creation of a continuous flow through the portal vein. Perfusate consisted of 4 l of UW Machine Perfusion Solution, supplemented with 3 mmol/l glutathione and was oxygenated at pressures above 450 mmHg, by two hollow fiber membrane oxygenators [45]. The study reported patient and graft survivals of 100% after 6 months and 1 year. Comparing outcomes with a matched cohort of 20 SCS DCD liver transplantations, peak serum ALT levels were significantly lower in recipients of D-HOPE-treated grafts (*p* = 0.006), as was serum bilirubin at POD7 (*p* = 0.044). No significant differences in intensive care unit (*p* = 0.475) or HLS (*p* = 0.88) were observed. Of note, 3 patients of the D-HOPE group developed post reperfusion hypokalemia (*p* = 0.03), without significant differences in other postoperative complications [45].

Recently, in 2018 and 2019, two further articles reported the use of D-HOPE in liver transplantation [43, 44]. Dondossola et al. utilized D-HOPE in 5 DCD grafts and 2 DBD grafts, which required prolonged preservation time [44]. Patrono et al. reported the use of D-HOPE in 4 cases of higher risk DBD liver transplantation either due to donor issues, severity of liver disease in the recipient, or both [43]. Both studies used the Liver Assist device for organ perfusion and D-HOPE was initiated after a preceding period of SCS [43, 44]. Dondossola et al. observed 3 cases of post reperfusion syndrome (PRS) with no cases of PNF. EAD occurred in 2 cases (1 DCD and 1 DBD graft). One DBD graft included was discarded after additional viability assessment through normothermic ex vivo liver machine perfusion (NEVLP). After a median follow-up of 270 (106–582) days, patient and graft survivals were 100% with no occurrence of biliary complications [44]. Similar outcomes were reported by Patrono et al. with patient and graft survivals of 100% at 6-month follow-up. There was no clinical evidence of ischemic cholangiopathy. In their trial, they experienced one case of post reperfusion syndrome and two cases of EAD [43].

### Normothermic ex vivo liver machine perfusion

NEVLP was first introduced into clinical practice through a Phase-I clinical trial performed by Ravikumar et al. in 2016 [51] and shortly followed by two Northern American studies published by Selzner et al. in 2016 and Bral et al. in 2017 [52, 53]. For organ perfusion, the portable OrganOx Metra® device was used in all studies, allowing normothermic perfusion of the portal vein and hepatic artery simultaneously [51–53]. Perfusate consisted of Gelofusine® in the trials of Ravikumar et al. and Bral et al. [51, 53], while Selzner et al. utilized Steen solution [52]. For oxygen carriage, three units of packed red blood cells were added to the perfusate in all trials [51–53]. Perfusion was initiated after organ retrieval and back table preparation at the donor center and not preceded by a period of SCS. DCD and standard DBD donors were eligible for inclusion in all studies, although the percentage of utilized DCD grafts was higher in the trial of Bral et al. (40% versus 20% in the studies of Selzner et al. and Ravikumar et al.) [51–53]. The primary objective of all studies was the evaluation of the safety and the feasibility of NEVLP in human liver transplantation. In total, NEVLP of 42 grafts was reported through these trials, of which 39 were successfully transplanted [51–53]. One graft was lost in the Bral et al. study due technical error (an obscure portal vein twist prevented successful NEVLP) [53] . Two grafts reported by Selzner et al. were discarded due to poor performance during NEVLP in the context of marginal donor characteristics or anatomic unsuitability for transplantation [52]. No case of PNF was reported throughout the trials with a 100% 30-day patient and graft survival [51–53]. Follow-up was limited by a three-month interval by Selzner et al. in which no case of biliary complication or graft failure was observed [52]. Bral et al. reported a 6-month follow-up, with no incidence of biliary complication and a patient survival of 89% [53]. Six-month and 1-year patient survival reported by Ravikumar et al. were 100% and 95%, respectively [51]. Intensive care unit stay and HLS of patients receiving NEVLP preserved grafts were not significantly different compared with matched SCS controls in the studies performed by Selzner et al. and Ravikumar et al. [51, 52], while they were longer in the trial performed by Bral et al. (ICU *p* = 0.004; HLS *p* = 0.01) [53]. Finally, Ravikumar et al. reported lower peak AST levels in patients undergoing transplantation of NEVLP preserved grafts compared with a SCS matched cohort (*p* = 0.034) [51].

In 2018, a landmark study in the field of NEVLP was published by Nasralla et al., reporting the first multicenter-randomized controlled trial, comparing NEVLP with SCS [54]. In this study, *n* = 334 liver grafts were randomized to either NEVLP or SCS, leading to the successful transplantation of 121 NEVLP and 101 SCS liver grafts. Seven transplant centers from four different European countries participated in the study. For graft perfusion, the OrganOx Metra® device was used. Of the included grafts, 37.1% were retrieved from DCD donors in the NEVLP arm and 36.6% in the SCS-arm [54]. The study met its primary endpoint regarding recipient peak AST levels post transplantation, showing a reduction of peak levels by 49.4% in the NEVLP group (*p* < 0.001). Subgroup analyses showed that the benefit of NEVLP, regarding peak AST, was higher for DCD grafts (*p* = 0.012). Compared with respective grafts of the SCS-arm, utilization of NEVLP reduced the geometric mean peak AST levels by 73.3% in DCD grafts (*p* < 0.001) and 40.2% in DBD grafts (*p* = 0.001). The odds reported for developing EAD were 74% lower in the NEVLP group (12 out of 119) compared with the SCS-group (29 out of 97) (*p* < 0.001), as were median serum Bilirubin levels (*p* = 0.029) [54]. No differences were reported regarding intensive care unit or HLS. Furthermore, 1-year patient and graft survival were similar, with patient survival of 95.8% vs. 97% and graft survival of 95% vs. 96% in the NEVLP and the SCS group, respectively [54]. One case of PNF occurred in the study and was in the NEVLP group. Notably, a significantly lower amount of discard was noted in the NEVLP arm compared with the SCS arm (*p* = 0.008), with longer median total preservation times in livers undergoing NEVLP (*p* < 0.001) [54].

Similar results, regarding the incidence of EAD and peak AST levels, were reported by a non-randomized phase one trial published by Liu et al. in 2020 [55]. The trial was carried out with the purpose of evaluating fresh frozen plasma as perfusate, along with the demonstration of safety and feasibility for the use of a non-commercial, institutional developed perfusion device [55]. NEVLP was performed in 21 liver grafts of which 38% were obtained from DCD donors. The perfusion device was carried to the retrieval site in 6 cases, while in the remainder, perfusion was started upon graft arrival at the transplant center in SCS. A maximum of 4 h cold ischemia time before NEVLP was limited by the study protocol [55]. Using a 1:4 historical matched cohort, post-transplant outcomes demonstrated a lower incidence of EAD in the NEVLP group (19% vs. 46%; *p* = 0.02) and lower levels of peak AST (*p* = 0.001), peak ALT (p = 0.001), and total Bilirubin on POD7 (*p* = 0.001) were reported in the NEVLP group. One-year patient survival was reported 95.23% [55].

Relevant obstacles for introduction of NEVLP in clinic practice included logistical challenges of perfusion device transport accompanied by trained staff, as well as higher material costs compared with SCS [4]. While hypothermic machine preservation approaches were applied end ischemic from their first introduction into clinic use [46, 47], NEVLP original premise was to avoid preceding periods of SCS, thus requiring transport of the NEVLP device to the organ retrieval sites [51–54]. Rationale for this approach was supported by a porcine study, showing inferior graft function when delaying initiation of NEVLP [56]. In order to assess the preservation benefit of NEVLP, when accompanied by a previous period of SCS, two trials were published in 2019 comparing immediate initiation of NEVLP with a pSCS-NEVLP approach [57, 58]: Bral et al. reported a single-center nonrandomized trial, in which 17 locally procured livers, with initiation of NEVLP immediately after graft retrieval, were compared with 26 livers retrieved from distant sites, with initiation of NEVLP after graft transportation to the study center in SCS [57]. The principle of initiating NEVLP after graft arrival at the transplant center was coined “back to base” and performed to allow easier complementation of NEVLP. In the trial, 10 (23%) grafts were obtained from DCD donors of whom 4 were in the local NEVLP and 6 in the “back-to-base” group. SCS times in the “back to base” group were significantly longer compared with the local NEVLP subjects (*p* = 0.001) with similar periods of NEVLP (*p* = 0.19). Total preservation times tended to be longer in the “back-to-base group” without reaching significance (*p* = 0.06) [57]. The primary outcome, 30-day patient and graft survival, was 100% in both groups. Furthermore, no significant difference regarding patient and graft survival at 3- and 6-month (*p* = 0.1), incidence of EAD (*p* = 0.29), peak levels of liver function parameters in the first postoperative week (AST *p* = 0.63; ALT *p* = 0.95; Bilirubin *p* = 0.43; INR *p* = 0.95), or biliary complications (*p* = 0.69) was observed [57]. Interestingly the intensive care unit and HLS were shorter in the “back to base” group (ICU *p* = 0.004; HLS *p* = 0.001). Comparing the overall experience of 43 NEVLP liver transplantations reported in the trial with a matched cohort of 86 SCS grafts, the group reported similar results to those seen previously [51, 54], observing significantly lower peak AST levels in the first post-operative week in patients undergoing transplantation after NEVLP (*p* = 0.04). Furthermore, NEVLP had the logistical advantage of having more transplantations during the daytime compared with the SCS controls (*p* = 0.04). [57].

In the same year, a separate trial investigating the feasibility of pSCS-NEVLP was published by Ceresa et al. [58]. In the multicenter study, pSCS-NEVLP was performed in 30 grafts obtained from DBD (74%) and DCD (26%) donors. This was compared with a cohort of 104 livers preserved by continuous NEVLP. The cases used for comparison were part of the NEVLP group, reported by Nasralla et al. in 2018. The study showed safety and feasibility for pSCS-NEVLP with regard to 30-day graft survival (94%) [58]. Furthermore, no significant differences in post-transplant outcomes, concerning serum peak AST levels (*p* = 0.92), incidence of EAD (*p* = 0.75), post reperfusion syndrome (*p* = 0.99), major complications (Clavien-Dindo ≥IIIb) (p = 0.99), and hospital (*p* = 0.88) or intensive care unit (*p* = 0.93) length of stay, were observed. One-year graft survival was 84% and similar to the control group (*p* = 0.08) [58]. Of note, similar to the report of Bral et al. [57], NEVLP once again demonstrated improvements of transplantation logistics with 71% of pSCS-NEVLP liver transplantations initiated during the daytime (8 a.m. to 8 p.m.) [58].

The clinical feasibility of the technique was further demonstrated by the Pisa (ITA) and the Innsbruck (AUT) group [59, 60]: Ghinolfi et al. used a pSCS-NEVLP approach in a randomized single-center trial comparing NEVLP with SCS for grafts from elderly donors [59]. In the study, 20 grafts retrieved from donors of 70 years of age or older were randomized to pSCS-NEVLP or SCS and eventually transplanted. The primary endpoint was 6-month patient and graft survival and showed non-inferiority of NEVLP preservation [59]. Although histological findings associated NEVLP with a reduction of IRI, no significant differences in clinical outcomes were observed [59]. Cardini et al. recently reported the introduction of routine use of pSCS-NEVLP for marginal donors, logistical challenges, and for complex recipients at the University Hospital of Innsbruck (AUT) [60]. The authors describe a multidisciplinary approach to NEVLP and the establishment of a 24/7 applicable clinical protocol. Analyzing the first 35 cases, the authors report a major improvement in logistics through prolongation of preservation times of up to 30 h, allowing reduction of simultaneous operations and omitting nighttime transplantations. Of the first 35 cases, 25 were transplanted with a patient survival of 88% at a mean follow-up of 8.6 (± 5.9) months. Of these, 90% of the grafts were ECD [60].

Compared with SCS and HMP approaches, NEVLP permits graft preservation in a metabolically active state, not only reducing ischemic times but also allowing for ex situ assessment of graft metabolism. Different suggestions for viability criteria have been made, although clinical evaluation is still pending [35–37, 61, 62]. To date, reported viability assessments during NEVLP is based on a holistic interpretation of different perfusion parameters such as lactate clearance, bile production, perfusate pH, glucose metabolism, flow rates, and perfusate transaminases. In addition to the findings of Nasralla et al., where NEVLP lead to a lower discard rate in grafts randomized to NEVLP [54], Cardini et al. reported that the possibility of graft evaluation via NEVLP led to increased consideration of grafts [60]. In 2016, Mergental et al. reported successful transplantation of five liver grafts declined by all the UK centers, after viability assessment (lactate clearance, bile production, perfusate pH, hepatic artery and portal vein flows, and homogeneity of graft perfusion) via NEVLP [36]. Patient survival was 100% after a 6–19 month follow-up, with no case of PNF reported [36]. A further trial investigating the use of NEVLP for viability assessment in high-risk ECD grafts was published by Watson et al. (2017) [37]. The trial reported transplantation of 12 livers assessed by NEVLP. Median donor risk index was 2.15 (1.47–3.14) with two grafts being allocated through an offer for research. Although the study reported adverse outcomes in their initial phase (observing post reperfusion syndrome in 5 of 6 grafts with one case of PNF), reevaluation of their perfusion protocol led to adjustments in oxygenation and allowed subsequent uneventful perfusions and graft evaluation which was uneventful [37]. Specifically, changes in lactate, glucose, and transaminase concentrations, as well as maintenance of perfusate pH, were used for viability assessment and led to 1-year graft and patient survivals of 83% and 92% respectively [37]. Most recently, Mergental et al. reported on the outcomes of the VITTAL clinical trial (ClinicalTrials.gov NCT02740608), in which declined liver grafts were assessed by NEVLP. Included grafts must have been declared unsuitable by all the UK transplant centers in addition to meeting one of seven predefined high-risk criteria (e.g., graft macrosteatosis > 30% or peak donor transaminases > 1000 U/ml). Viability assessment was based on lactate clearance below 2.5 mmol/l within the first 4 hours of NEVLP alongside fulfillment of two or more further criteria, such as bile production, perfusate pH ≥ 7.3, metabolism of glucose, HA flow ≥ 150 ml/min and PV flow ≥ 500 ml/min, or homogenous perfusion. Of 31 assessed grafts, 22 met criteria and were eventually transplanted reaching 100% 90-day patient and graft survival [63].

### Sequential machine perfusion

While hypothermic machine preservation targets consistent graft temperatures below 10 °C and NEVLP aims to keep perfusion at physiologic body temperatures, a more dynamic approach towards modulating perfusion temperatures has also been utilized: In 2016, Hoyer et al. demonstrated safety and feasibility for controlled oxygenated rewarming (COR) in a series of six liver transplantations [64]. In this study, SCS grafts were assigned to oxygenated, dual-vessel, ex vivo machine perfusion, for 90 min preceding implantation. Machine perfusion was used to slowly warm grafts before implantation by raising perfusate temperatures from 10 °C to 20 °C throughout perfusion. Patient and graft survival were 100% after 6-month follow-up and COR grafts demonstrated, decreased levels of peak transaminases in the post-operative period (AST *p* = 0.023; ALT *p* = 0.038) [64]. Recently, the group published long-term outcomes of grafts treated with COR preceding transplantation, extending the initial series by 12 patients up to a total of 18 cases, and reporting 1-, 3-, and 5-year patient survival rates of 100%, 100%, and 93.8%, respectively [65]. A trial published by van Leeuwen et al. in 2019 further aimed to combine the beneficial features of D-HOPE and NEVLP for graft resuscitation and viability assessment, in a study of nationwide declined grafts [35]. In the trial HBOC-201, a hemoglobin-based, cell free oxygen carrier was used for substitution of red blood cells, allowing uninterrupted transition from a D-HOPE phase over a period of COR to NEVLP. Grafts were transported in SCS to the study center and evaluated during NEVLP. Grafts that met predefined viability criteria (perfusate lactate< 1.7 mmol/L; pH 7.35 to 7.45; bile-production > 10 mL; bile pH >7.45) were subsequently transplanted [35]. Of the 16 perfused livers studied, 11 ultimately met viability criteria and thus were transplanted, with 100% patient and graft survival at 3 and 6 months, respectively. All transplanted grafts were obtained from DCD donors with a median Eurotransplant donor risk index (ET-DRI) of 2.82 (2.6–2.9). During the study period, graft evaluation by machine perfusion led to an increase of deceased donor liver transplantation by 20% in the study center [35].

### Ongoing clinical trials

Currently, 12 studies investigating the role of machine perfusion in liver transplantation are enrolled in the ClinicalTrials.gov registry. There are a greater number of studies using a normothermic approach (*n* = 7) compared with those using hypothermic perfusion (*n* = 4). Sequential hypothermic to normothermic machine perfusion is being investigated in one single-center prospective pilot study, registered by the Cleveland Clinic (Ohio, USA) group. Of the hypothermic approach, two are randomized multicenter trials, of which one uses regular liver grafts, while the other focuses on ECD DBD grafts. Regarding currently active studies investigating NEVLP, the objective of all except one is the assessment of the effect of NEVLP vs. SCS on ECD liver grafts. Interestingly, two of the enrolled trials target assessment and resuscitation of declined liver grafts by NEVLP, applying viability criteria such as lactate clearance and bile production.

## Discussion

Over the last 5 years, the burgeoning number of published and ongoing clinical trials investigating ex vivo machine perfusion in liver transplantation reflects a renewed interest of this platform technology. Challenging the long-time paradigm of static cold storage of liver grafts in ice boxes, ex vivo liver machine perfusion holds promise as a method to increase the safety and utilization of marginal livers for transplantation. This might be achieved not only through viability assessment of high-risk grafts but also through reconditioning of otherwise unusable liver grafts.

Review of the current literature suggests that ex vivo liver perfusion, at hypothermic as well as normothermic perfusate temperatures, reduces preservation injury in standard criteria grafts. This observation is supported by a reduced incidence of EAD and decreased levels of peak transaminases. However, whether machine perfusion of a standard graft is necessary remains to be answered as no mortality benefit was noted compared with patients undergoing transplantation of standard criteria SCS liver grafts. Due to its high costs, application for pre-defined indications might facilitate adoption and maximize the benefit of ex vivo liver machine perfusion in broader clinical practice. As reported by the Zürich group, HOPE allowed reintroduction of DCD liver transplantation, showing improved outcomes regarding intrahepatic cholangiopathy compared with matched SCS DCD grafts and thus expanded their donor pool [48]. Although data from randomized controlled trials on HOPE have not been reported as of yet, current literature suggests its superiority towards classic SCS, raising the question as to whether this technically and logistically less demanding technology (compared with NEVLP) might evolve for routine use in standard criteria donors, especially in cases with expected prolonged hepatectomy times.

An increasing number of studies reported application of ex vivo machine perfusion in extended criteria donors and suggests beneficial effects regarding EAD may be achieved by both the hypothermic and normothermic approach. The Zürich group observed better outcomes in a series of HOPE-treated steatotic grafts, when compared with a cohort of SCS fatty liver transplantations [50]. Furthermore, the Pisa group reported histological evidence of reduced IRI after NEVLP of grafts from donors older than 70 years of age in a single-center randomized controlled trial [59]. Although the evidence for positive effects of machine perfusion on marginal grafts is still limited, the interest continues to grow as reflected by the number of ongoing clinical trials. Given the current demographic change in the western world and the projected dramatic incidence of hepatic steatosis in the donor pool, ex vivo liver machine perfusion might be an ideal tool for preservation of marginal liver grafts prior transplantation. [66, 67].

To fully exploit the potential of ex vivo machine perfusion for marginal grafts, further insight in the underlying mechanisms of the effects of machine perfusion on liver physiology and metabolism remains paramount. For instance, Lai et al. concluded in a systematic review, assessing ex vivo liver machine perfusion before transplantation in 54 cases of grafts affected by macrovesicular steatosis (24% showing moderate to severe steatosis [≥ 30%]) that no differences in clinical outcome such as post-transplant death or severe complications following machine perfusion could be identified. Raigani et al. have demonstrated that steatotic liver grafts suffer from deficits in antioxidant capacity, efficient energy utilization, and lipid metabolism during normothermic liver machine perfusion. [68]. Thus, although the near physiologic state maintained by NEVLP is beneficial in and of itself, it may additionally serve as a platform to allow further optimization of marginal liver grafts by drug application during ex vivo machine perfusion. Currently, several groups are working on protocols for “defatting” of steatotic liver grafts, with the idea of decreasing the detrimental effect of IRI on these organs after reperfusion [69–71]. Indeed, a recently published report of the Zürich group, preserving injured human livers for 7 days by ex vivo machine perfusion, encourages research towards drug application as the available period for ex vivo drug treatment is significantly prolonged [72]. It must still be evaluated if further marginality criteria, e.g., extended age of the donor, might also be addressed during ex vivo machine perfusion. So far, it is unknown whether these organs have specific needs that could be met during perfusion or not. The currently ongoing trials will hopefully provide insight of the impact machine perfusion has on marginal liver grafts by providing not only clinical data but also biologic samples to address those questions. Beyond these applications, machine perfusion offers even broader possibilities such as HCV clearance with existing drugs as has already been proposed experimentally [73]. Transfection with viral vectors or siRNA may also allow for immunomodulation and reduced immunosuppression [74, 75]. Infusion of mesenchymal stem cells can ameliorate preexisting damages such as warm ischemia [76]. Even genetic modulation during machine perfusion may lead to a new frontier in the field of transplantation medicine and advanced treatments allowing for engineered or personalized grafts. As the drugs or agents are applied ex vivo, they are unaffected by the humoral and cellular immune system and can be washed from the liver at the end of perfusion, thus allowing for potential use of treatments not feasible in vivo.

When comparing hypothermic and normothermic machine perfusion approaches, it is important to note that normothermic systems enable metabolic characterization and viability assessment of the grafts by “simple” read out parameters like bile production, bile pH, or lactate clearance, while reduced metabolic activity during hypothermia restricts the viability assessment capacity of HMP systems. Although specific surrogate parameters or biomarkers might be able to fill this gap, as shown by Muller et al. [77], normothermic approaches simulate near physiologic conditions allowing for use of classic clinical parameters for graft evaluation. A European multicenter-randomized controlled trial showed significantly lower discard rates in grafts randomized to the NEVLP arm [54]. Moreover, van Leeuven et al. and Mergental et al. reported “resuscitation” and successful transplantation of declined liver grafts after back-to-base machine perfusion. Assessing pre-defined viability criteria during the normothermic perfusion phase, both groups achieved high 1-year graft survivals of livers initially declined for transplantation [35, 63].

Lastly, as success in transplantation remains dependent on multidisciplinary care, decreasing strain on treatment teams remains important. By allowing for prolonged preservation periods up to 30 h [60], NEVLP has improved operating room logistics by permitting more daytime transplantations as reported by three groups [57, 58, 60]. Inherently, this provides for increased availability of experienced staff across disciplines, [60] and has the potential to further optimize outcomes and decrease failure to rescue after liver transplantation.

In line with our conclusions, the Italian Society of Organ and Tissue Transplantation (SITO) recently released their evidence-based position paper on machine perfusion in liver transplantation [78], recommending the consideration of HMP and NEVLP as safe techniques for organ preservation and suggesting its use for reduction of post-transplant EAD. Furthermore, it recognized hypothermic- and normothermic-machine perfusion as useful tools for prolongation of ex vivo times and improvement of transplantation logistics. HMP was suggested as useful for improvement of graft survival in ECD and DCD donors and for reduction of ischemic type biliary lesions. NEVLP was considered useful for the implementation of the use of ECD donors, by allowing evaluation of graft function and highlighting the potential of NEVLP for ex vivo viability assessment [78].

In conclusion, ex vivo liver machine perfusion has significant potential to revolutionize the field of liver transplantation. In a field challenged by a persistent organ shortage and continued donor demographic changes threatening the quality of the already scarce pool of available grafts, the use of this technology may help in addressing the daunting waitlist mortality. Within its first decade of clinical introduction, it has proven to be safe and feasible, for hypothermic and normothermic perfusate temperatures, and demonstrated improved early graft function, compared with SCS preserved grafts. These findings have allowed reintroduction of DCD programs in countries with long mandatory no touch periods and salvage of grafts that otherwise would have been discarded. Considering the early stage of the technology, it remains critical to gain further insight into the underlying mechanisms that lead to observed clinical change, and the possibility of expanding use of this technique for treatment and reconditioning of otherwise unusable donor grafts.

## Abbreviations

EAD: Early allograft dysfunction
PNF: Primary nonfunction
DBD: Donation after brain death
DCD: Donation after circulatory death
IRI: Ischemia-reperfusion injury
SCS: Static cold storage
HMP: Hypothermic machine perfusion
HOPE: Hypothermic oxygenated machine perfusion
D-HOPE: Dual hypothermic oxygenated machine perfusion
HLS: Hospital length of stay
pSCS: post-Static cold storage
NEVLP: Normothermic ex vivo liver machine perfusion
